# Pushing the Boundaries of Minimally Invasive Surgery: Fully Laparoscopic Left Hepatectomy Extended to Segment 8 for Bilobar Colorectal Liver Metastases

**DOI:** 10.7759/cureus.74557

**Published:** 2024-11-27

**Authors:** Camila Sotomayor Ledezma, Natalia Reyes, Pedro Soto, Eduardo Briceño, Martín Dib, Eduardo Viñuela, Jorge Martínez, Nicolás Jarufe

**Affiliations:** 1 Department of Hepatobiliary and Pancreatic Surgery, Pontificia Universidad Católica de Chile, Santiago, CHL; 2 Department of Surgery, Hospital Clínico Dra. Eloísa Díaz I. La Florida, Santiago, CHL; 3 Division of Transplantation, Department of Surgery, Beth Israel Deaconess Medical Center, Harvard Medical School, Boston, USA

**Keywords:** colorectal liver metastasis, laparoscopy, left extended hepatectomy, major hepatectomy, minimally invasive surgery

## Abstract

The surgical management of hepatic metastases from colorectal cancer may range from segmental resections to major or extended hepatectomies. The aim is to achieve complete removal of metastatic lesions while preserving adequate liver function.

We present the case of a 42-year-old male patient with a history of glucose intolerance who presented with altered bowel movements and abdominal pain. After further evaluation, he was diagnosed with stage IV rectosigmoid cancer with potentially resectable bilobar liver metastases in segments 2, 4a-8, and 6. KRAS, NRAS, and BRAF were wild-type, and no microsatellite instability was detected.

The patient underwent six cycles of chemotherapy with FOLFOX (oxaliplatin in combination with 5-fluorouracil and leucovorin), and radiofrequency ablation (RFA) was applied to the lesion in segment VI, resulting in a favorable response in imaging control. Consequently, we perform a laparoscopic extended left hepatectomy with wedge resection of the segment VI lesions previously treated with RFA.

The video shows a completely laparoscopic left hepatectomy extended to segments 5 and 8 and also a resection of S6 metastasis. It is possible to appreciate the management of the left hepatic pedicle and the transection of the parenchyma with the use of energy instruments: cavitron ultrasonic surgical aspirator and bipolar. In addition to the dissection and section of the middle and left hepatic vein included in the surgical specimen.

The patient experienced a rapid postoperative recovery with good liver function, an early hospital discharge, and a quick return to work, highlighting the clear advantages of laparoscopic surgery.

## Introduction

Colorectal liver metastases (CRLM) are a significant cause of mortality in patients with cancer, particularly colorectal cancer, which affects over 1.3 million individuals globally each year [1]. It is estimated that 50-60% of patients diagnosed with colorectal cancer will develop CRLM, and among these, 80-90% present with unresectable metastatic liver disease [1].

The surgical management of CRLM is a cornerstone in the treatment of patients with isolated liver metastases [2]. Surgical resection remains the most effective and potentially curative option for CRLM, aiming for complete removal of metastatic lesions (R0 resection, defined as negative microscopic margins). This requires maintaining at least two disease-free liver segments with intact portal and arterial inflow, as well as venous and biliary outflow, and a sufficient functional liver remnant (FLR) to sustain postoperative liver function [3].

Surgical interventions range from minor segmental resections and major or extended hepatectomies to liver transplantation, depending on the size, number, and location of metastases [4].

Over the past two to three decades, advancements in systemic therapies, surgical techniques, and local treatments have significantly improved survival rates for patients with liver metastases. Specifically, systemic chemotherapy and biologic agents have enabled the downsizing of some initially unresectable CRLM to resectable disease, expanding treatment possibilities [1].

Despite these advancements, only approximately 20% of patients with isolated CRLM are eligible for surgical resection. Factors such as disease burden, tumor location, liver functional reserve, and the patient’s overall functional status often preclude surgery. For these patients, non-resectional therapies, including radiofrequency ablation (RFA) and microwave ablation, are valuable alternatives that may achieve curative outcomes [3].

Surgical resection is considered the gold standard. The continuous evolution of surgical techniques over the last decade has further improved the outcomes of liver resections, reinforcing their critical role in the multidisciplinary management of CRLM [2].

This case report was previously presented as a meeting video at the 2024 IHPBA World Congress, held in Cape Town, South Africa, on May 15-18, 2024.

## Case presentation

We present the case of a 42-year-old male patient with a history of glucose intolerance who presented with altered bowel movements and abdominal pain. A colonoscopy revealed a stenotic tumor in the rectosigmoid area, with a biopsy positive for moderately differentiated tubular adenocarcinoma, KRAS, NRAS, and BRAF wild type. There was no microsatellite instability. The carcinoembryonic antigen (CEA) level was 11 ng/mL (Table 1).

**Table 1:** CEA level at diagnosis CEA: carcinoembryonic antigen

Test	Results	Reference range
CEA level at diagnosis	11 ng/mL	Non-smokers: <3.8 ng/mL, smokers: <5.5 ng/mL

**Figure 1:**
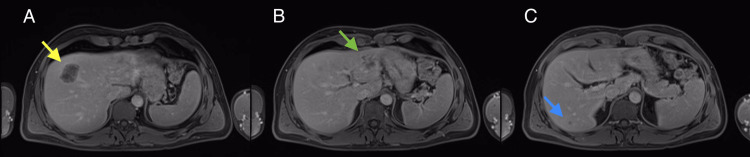
MRI axial plane A: Lesion at the interface of segments 8/4a. B: Lesion in segment 2. C: Lesion in segment 6. MRI: magnetic resonance imaging

Magnetic resonance imaging (MRI) (Figure 1A-1C) showed four hepatic metastases: 20 mm in segment 2, 39 mm at the interface of segments 4a and 8, 7 mm in segment 6, and 11 mm in the lower pole of segment 6. Additionally, a follow-up was performed with a post-chemotherapy anorectosigmoidoscopy, and tattooing was performed with two spots distal to the tumor.

**Figure 2:**
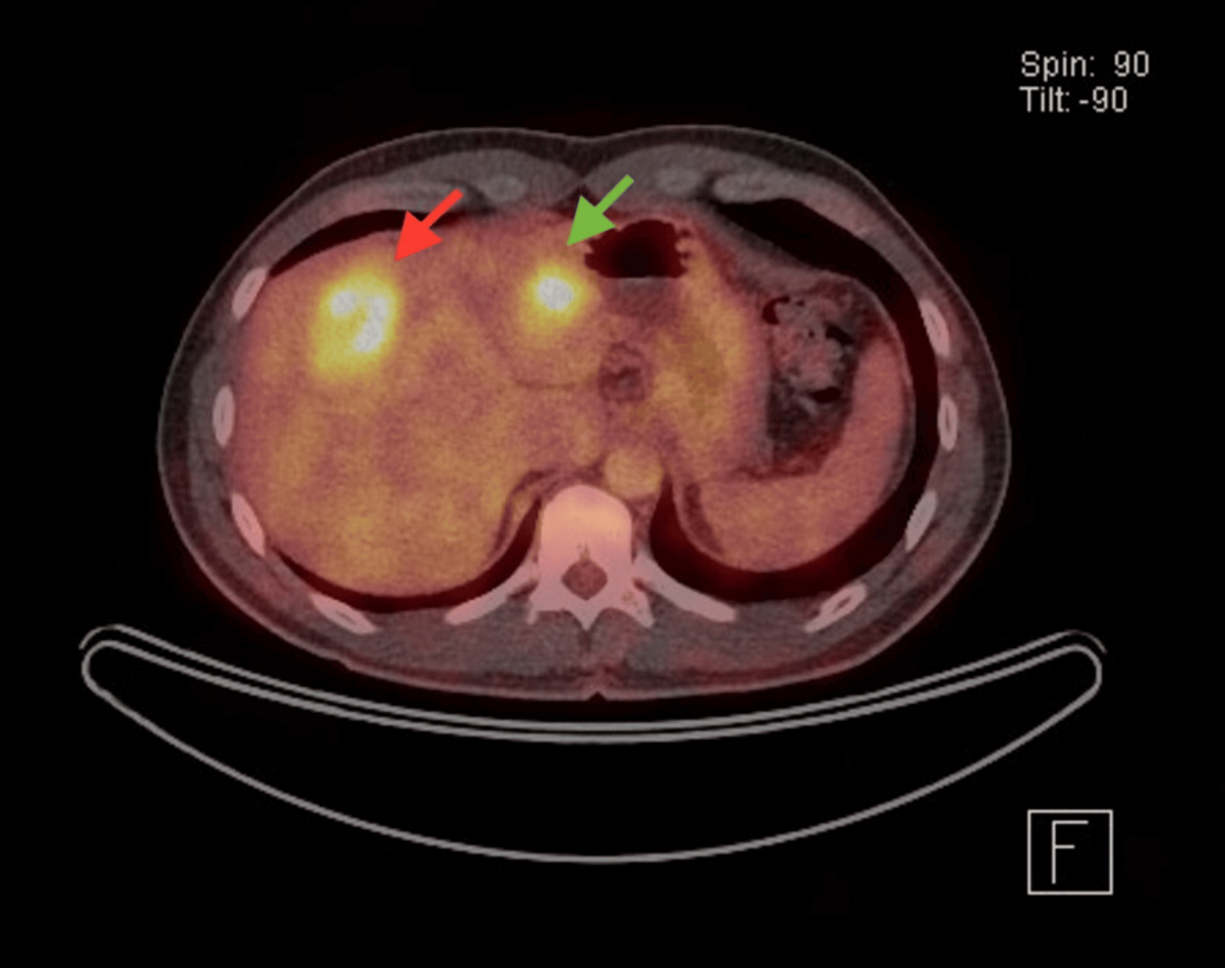
Initial PET-CT axial plane Red arrow: Lesion in segment 8-4a. Green arrow: Lesion in segment 2. PET-CT: positron emission tomography-computed tomography

**Figure 3:**
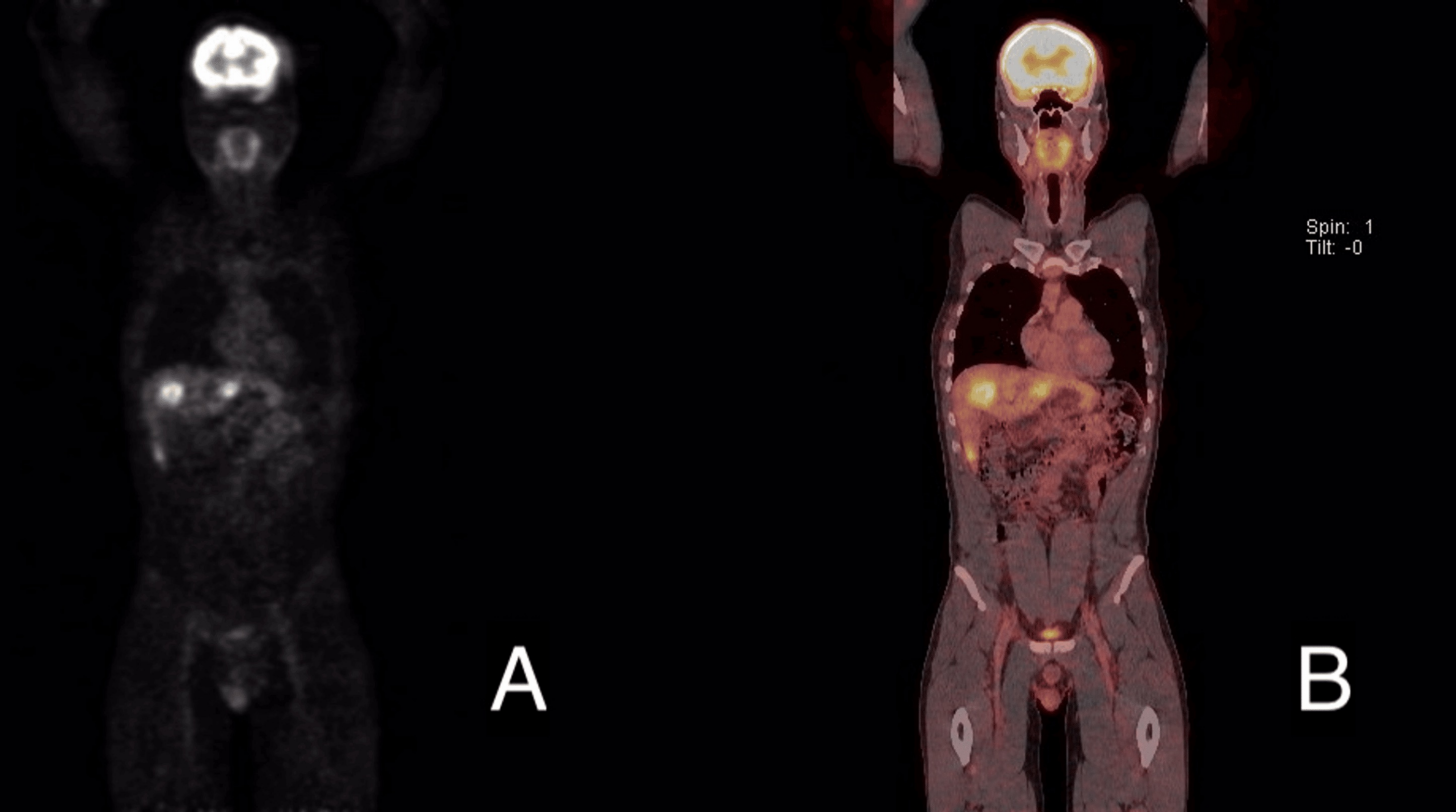
Initial PET-CT coronal plane A: PET image acquisition phase. B: Fusion of PET and CT images. Hypermetabolic bilobar liver metastases are visible in both images, located in segments 8/4a, 2, and 6. PET-CT: positron emission tomography-computed tomography

Further evaluation with a PET-CT scan (Figures 2-3) showed the previously known transmural tumor in the upper rectum with increased uptake (SUV max 8.12), and at least three potentially resectable bilobar liver metastases: the largest measuring 42 mm in segment 8-4a (SUV max 6.04), 22 mm in segment 2 (SUV max 6.11), and 14 mm in the lower pole of segment 6 (SUV max 3.73). Additionally, a small focal lesion in segment 7 was clearly visible on CT but only mildly hypermetabolic (SUV max 2.58). There was no extrahepatic disease.

**Figure 4:**
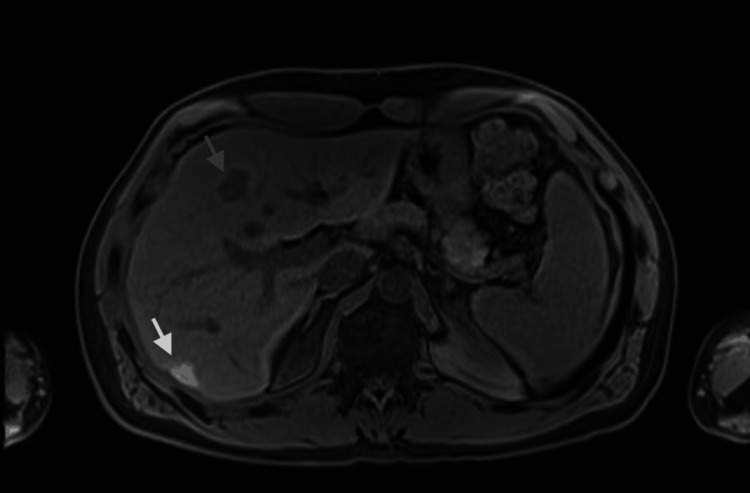
Follow-up MRI axial plane Gray arrow: Metastasis at the interface of segments 8/4a with a reduction in size from 40 to 26 mm. White arrow: In the posterior segment, at the site where a secondary lesion was previously visualized, there is a focus of T1 hyperintensity of 18 mm, secondary to coagulative necrosis transformation post-RFA. No foci of tumor viability were detected within it. MRI: magnetic resonance imaging, RFA: radiofrequency ablation

The patient received chemotherapy with a FOLFOX regimen (oxaliplatin in combination with 5-fluorouracil and leucovorin). After six weeks, RFA was applied to the lesion in segment 6. Three months after chemotherapy, there was a partial response with a reduction in the size of liver metastases, as seen on follow-up MRI (Figure 4): from 40 mm to 26 mm in the lesion located at the interface between the anterior and medial segments, and from 23 mm to 13 mm in the lesion in segment 2. In the posterior segment, at the site where a secondary lesion was previously visualized, there was a focus of T1 hyperintensity measuring 18 mm, secondary to coagulative necrosis transformation post-RFA of the known metastasis. No foci of tumor viability were detected within it.

Consequently, the patient underwent liver surgery. We performed a laparoscopic extended left hepatectomy plus metastasectomy of segment 6 lesion previously treated by RFA.

Video 1 shows a completely laparoscopic left hepatectomy extended to segments 5 and 8, plus a resection of S6 metastasis. It is possible to appreciate the management of the left hepatic pedicle and the transection of the parenchyma using energy instruments: cavitron ultrasonic surgical aspirator and bipolar, in addition to the dissection and section of the middle and left hepatic vein included in the surgical specimen.

**Video 1:** Fully laparoscopic left hepatectomy extended to segment 8 plus a resection of S6 metastasis

The patient had a rapid postoperative recovery with good liver function, early hospital discharge, and a fast return to work.

A biopsy of the left hepatic lobe showed an intraparenchymal CRLM of moderately differentiated tubular adenocarcinoma, measuring 1.8 cm, with 30% viable tumor and a negative surgical margin (0.6 cm from the edge). The round ligament and gallbladder were negative, and in segment 6 biopsy, a subcapsular hepatic metastasis of moderately differentiated tubular adenocarcinoma was found, measuring 0.5 cm, with a 30% viable tumor and a negative surgical margin.

**Figure 5:**
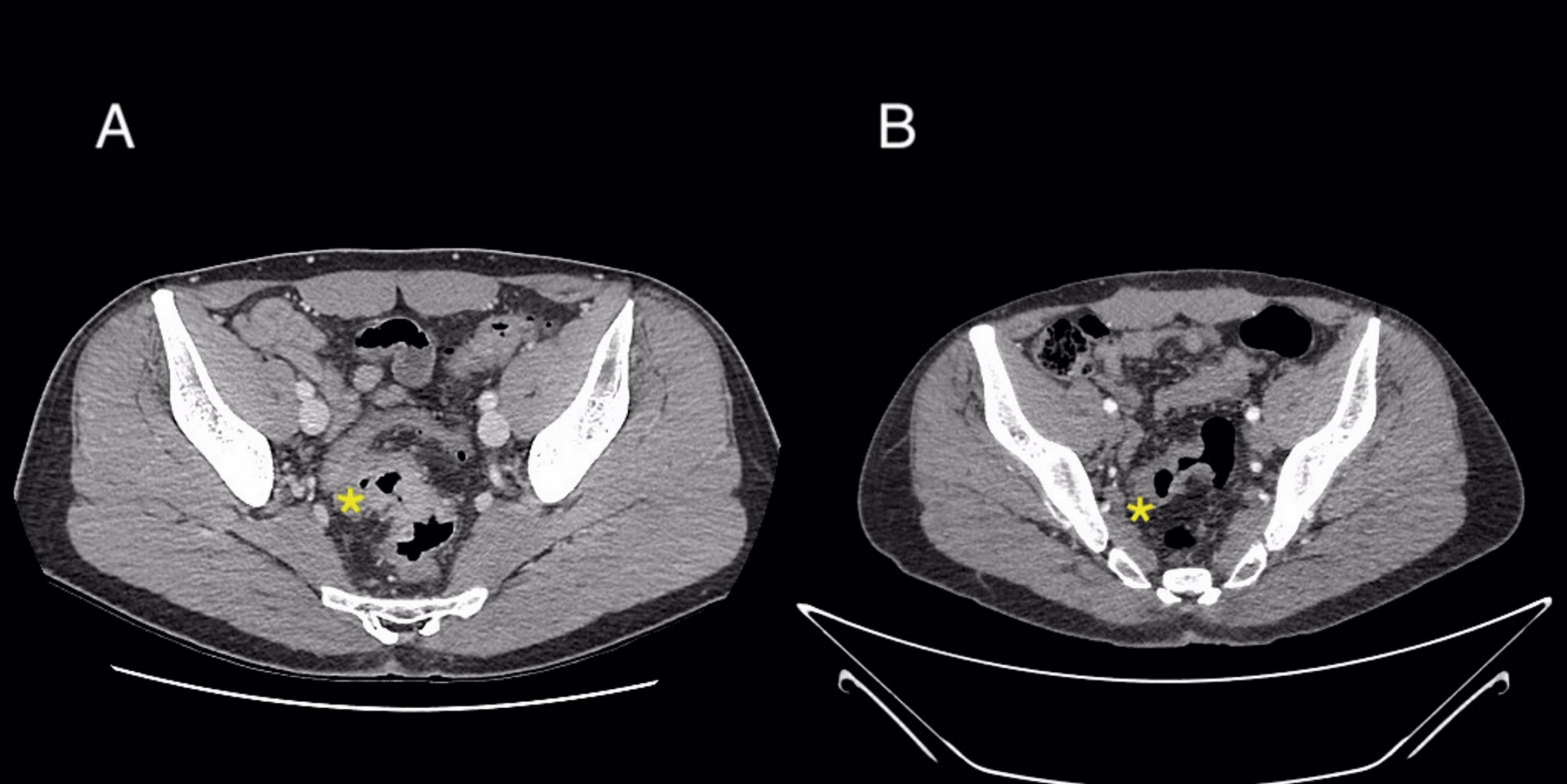
CT scan axial plane A: Prior to chemotherapy treatment. B: Following chemotherapy treatment. CT scan showed a significant reduction in the thickness of the parietal thickening of the rectosigmoid, now measuring 6 mm compared to 9 mm. CT: computed tomography

The patient completed three months of adjuvant FOLFOX. CT scan (Figure 5) showed a significant reduction in the thickness of the parietal thickening of the sigmoid colon, now measuring 6 mm compared to 9 mm in the previous CT scan.

**Figure 6:**
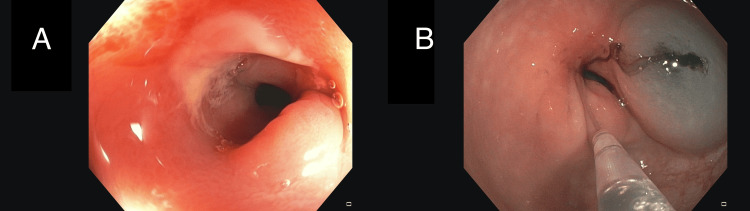
Post-chemotherapy anorectosigmoidoscopy A: At approximately 18 cm from the anal verge, a non-critical but impassable stenotic area was observed. This appeared to be a scar from a previously known tumor, with only angiodysplasia in the mucosa and no evidence of tumor viability. B: Tattooing was performed with two spots distal to the tumor.

Additionally, a follow-up was performed with a post-chemotherapy anorectosigmoidoscopy (Figure 6).

One year after the initial diagnosis and five months after hepatectomy, a sigmoidectomy was performed to resect the primary tumor. Biopsy results showed a moderately differentiated tubular adenocarcinoma measuring 2 cm, with three out of 21 lymph nodes positive, negative margins, no permeations, and a regression grade of 1. The pathologic staging was ypT3N1b.

At follow-up, six months after surgery, the patient is in good general condition, asymptomatic, with an Eastern Cooperative Oncology Group performance status of 0, normal liver function, and a CEA level of 2.4 ng/mL (Table 2).

**Table 2:** CEA level at follow-up CEA: carcinoembryonic antigen

Test	Results	Reference range
CEA level at follow-up (six months after primary tumor resection)	2.4 ng/mL	Non-smokers: <3.8 ng/mL, smokers: <5.5 ng/mL

## Discussion

Total laparoscopic liver resection (LLR) has gained acceptance in recent years due to its minimally invasive nature, which is associated with reduced postoperative pain, shorter hospital stays, and quicker recovery times compared to open surgery [5,6]. The feasibility and safety of laparoscopic minor liver resections are well established [7]. However, performing major hepatectomies in a minimally invasive manner requires advanced laparoscopic skills and experience due to the complexity of the procedure, including vascular control, parenchymal transection, and anatomical variation [8].

Extended hepatic resections, classified according to the 2000 Brisbane terminology, can be divided into two main categories: extended right hepatectomy and extended left hepatectomy.

Extended left hepatectomy involves the resection of segments 2, 3, 4, 5, and 8. Additionally, segment 1 may be partially or completely included in either the right or left extended resection [2,9], and when combined with metastasectomy of segment 6 (S6), it presents a challenging case, particularly in the laparoscopic setting. This approach is indicated when multiple metastases are confined to these liver segments and a sufficient FLR can be preserved. The decision to perform such an extensive resection laparoscopically reflects the evolving standards of care in hepatobiliary surgery, where minimally invasive techniques are increasingly being applied to complex liver surgeries without compromising the margins and therefore the oncological prognosis.

This case report describes the successful total laparoscopic extended left hepatectomy with S6 metastasectomy in a patient with CRLM. The case highlights the technical considerations, intraoperative challenges, and postoperative outcomes, contributing to the growing evidence supporting laparoscopic approaches in major liver resections.

The surgical technique employed is undoubtedly characterized by its great complexity, presenting a significant challenge for both surgeons and healthcare systems. This minimally invasive approach is associated with a long learning curve, requiring highly trained and experienced surgeons to complete the procedure, typically in specialized reference centers.

Beyond the technical complexity, several other factors contribute to the challenges of LLR. Vascular control during the procedure poses an increased operative risk, necessitating the use of highly sophisticated surgical technology and expensive equipment [10]. The requirement for this specialized equipment, coupled with the need for extensive surgeon training, can limit the accessibility of this approach [11].

Despite these challenges, advancements in surgical techniques and technology have led to improved outcomes for patients undergoing LLR. While in the beginning, mainly benign tumors were laparoscopically operated on, liver metastasis and hepatocellular carcinoma are now among the most frequent indications [12].

Various studies have demonstrated that laparoscopic resections offer favorable oncologic outcomes for HCC and CRLM, and their precise technique makes them a promising therapeutic option for liver malignancies [13]. While most of the available evidence comes from specialized centers in Asia, Europe, and the USA, a recent multicenter study from South America has shown an increasing number of centers performing LLR with promising perioperative results comparable to those of leading centers worldwide [14].

Ongoing research and training efforts are essential to address the unique complexities of this procedure and ensure the continued expansion of this minimally invasive approach to liver surgery [14].

## Conclusions

In this case, the successful total laparoscopic extended left hepatectomy combined with a metastasectomy of segment 6 demonstrates the feasibility and efficacy of laparoscopic techniques in the management of complex CRLM. The patient’s rapid postoperative recovery, with good liver function and early hospital discharge, underscores the benefits of a minimally invasive approach, which offers reduced postoperative morbidity and a faster return to normal activities. The R0 resection achieved, combined with adjuvant chemotherapy, contributed to a favorable oncological outcome, as evidenced by the patient’s good general condition and normal CEA levels during follow-up.

This case further supports the growing evidence that laparoscopic approaches can be safely extended to major hepatectomies, even in cases of bilobar metastases, provided that the procedure is performed by experienced surgeons in specialized centers. The challenges of vascular control, parenchymal transection, and anatomical variations are significant but can be overcome with advanced laparoscopic skills and the availability of specialized equipment. Continued advancements in surgical technology and ongoing training are essential to increase the accessibility and safety of these complex procedures, improving outcomes for patients with CRLM.
